# Commentary: an industry perspective on the importance of incorporating participant voice before, during, and after clinical trials

**DOI:** 10.1186/s13063-022-06905-6

**Published:** 2022-11-28

**Authors:** N. Goodson, P. Wicks, C. Farina

**Affiliations:** 1 THREAD Research, 155 El Camino Real, Tustin, CA 92780 USA; 2 Wicks Digital Health Ltd, Advantage House, Lichfield, Staffordshire WS13 6AQ UK

**Keywords:** Patient and public involvement, Recruitment, Retention

## Abstract

It is increasingly recognized that involving patients and the public in the design of clinical trials can lead to better recruitment, retention, and satisfaction. A recent scoping review determined that between 1985 and 2018, just 23 articles meeting quality criteria obtained feedback from clinical trial participants after a trial had been completed. In a timespan that presumably included thousands of trials across hundreds of indications, the paucity of the literature seems surprising, if not outright disappointing. By contrast, practitioners in the life sciences industry are increasingly incorporating patient research into their trial design process before, during, and after trial completion. Examples of approaches used include recruitment of “look alike” participant samples through online communities, surveys, and the use of smartphone apps to directly record participants’ spoken reactions to trial materials like recruitment materials, site visit schedules, or informed consent materials. However, commercial organizations tend not to publish their findings, leading to a potential two-tier experience for trial participants depending on whether the trial they participate in will be industry-funded or government-funded. This seems problematic on a number of levels. Increasing regulatory, funder, and publisher interest in improving the inclusivity of clinical trial participants may act as a timely lever to spur patient-centered coproduction of trials. Until continuous feedback processes are the mandated, funded, and published norm, participating in a clinical trial will be more arduous than it needs to be.

Most clinical trials lack any meaningful input from potential participants. This paucity of participant-centered design means trials can often be confusing, burdensome, and expensive for participants, leading to challenges with recruitment, retention, and missing data. In a recent scoping review in Trials by Signorell et al. [1], the authors reported that from a 33-year period there were just 23 studies in the peer-reviewed literature gathering retrospective feedback from participants *after* trials had been completed. Another review of experience gathered *during* the trial itself found just 22 studies published over 17 years [2] and input collected *before* a trial design was finalized was rarer still [3].

This is a shame, because as applied clinical trial researchers working in industry, such sparsity in the scientific literature stands in contrast to our commercial experience over the past decade. One of us (PW) co-authored a study of 1621 patients on the online community PatientsLikeMe [4] which was cited in the Signorell et al. [1] review. Our survey served as a pilot for approximately 25 study-specific (but sadly unpublished) studies conducted between 2014 and 2019 on behalf of trial sponsors or contract research organizations (CROs) which each followed a similar format [5]. First, a trial protocol was condensed into about a dozen patient-facing elements that could be shown in a survey. For example, the aims of the study, duration, number of site visits required, any invasive procedures, lifestyle changes required for the duration of the study, and availability of study medication after trial completion. Second, individuals with the same disease (e.g., multiple sclerosis, lupus, diabetes) were sent a survey invitation through online patient communities or online advertising. Third, these “look-alike” participants were invited to share their experiences of prior trials, interest in taking part in trials in general, and were then presented with each patient-facing trial element in turn with a single question “Does this element of the trial *increase* or *decrease* your interest in taking part?” along with an open text box for them to explain why.

In response to this mixed-methods feedback, we were pleased to find sponsors removing painful study procedures of minor scientific relevance, adding open-label extension studies, permitting the option of at-home questionnaire completion, and the addition of relevant patient-reported outcome domains such as pain and fatigue in more detail than previously planned. Sponsors were keen to reduce participant burden while maintaining high-quality data for regulatory purposes and also benefitted from a trial more likely to succeed [6]. While satisfying work, it is somewhat alarming that a single commercial research vendor may have acquired more patient experience feedback in 5 years than more than three decades of peer-reviewed scientific literature. The gap may be partially explained as a lack of published data. Some researchers do seek insights from the patient community prior to completing a trial, but those insights do not always reach peer-reviewed publications. But failure to publish alone does not fully explain the limited data.

Previous work from both industry and academia has shown that effective patient engagement decreases time to product launch and improves enrollment and retention [7, 8]. So why isn’t this routine? Despite the opportunity for benefit, obtaining input from real patients in a cost-effective, scalable, and study-specific manner can present real challenges to teams creating and operationalizing clinical trials. Even a simple patient panel can take significant time and cost to assemble and may not be pragmatic in many circumstances. An alternative approach we have been using to address this gap is voice-response technology. Here again, “look alike” participants who match the inclusion/exclusion criteria for a given study are recruited to review materials (e.g., a protocol or informed consent form), and speak their feedback directly into their smartphone or web browser. Their spoken words and tone are quantified using voice processing software, machine learning acoustic tools, and linguistic analysts to provide qualitative feedback about the study design, areas of strength, and potential improvements. Insights include ‘what was said’ via content and thematic analyses and “how it was said” to uncover underlying emotions, thoughts, and perceptions that drive behavior. The whole process typically takes less than 3 weeks. Anecdotally, we (NG, CF) have also found that sponsors and trialists who hear suitably deidentified recordings of prospective participants’ voices may engage more with the audio feedback than simply viewing graphs and pull quotes from survey responses.

A range of options are needed for industry to increase the frequency and consistency in participant feedback before, during, and after trials. To this end, the non-profit organization TranCelerate Biopharma recently developed [9], validated, and cross-culturally adapted the freely-available Study Participant Feedback Questionnaire (SPFQ) [10]. Fielding such tools as electronic surveys to participants at home is convenient, acceptable, and may reduce concerns about social desirability bias as compared to administration at site visits [1]. Similarly, the global multistakeholder Patient Focused Medicines Development group has shared their own playbook of best practice [11]. This includes “how-to” guides across phases of clinical development, practical considerations for working with patient stakeholders, and signposting to additional resources to engage further with participants. We are also aware of a number of pharmaceutical companies who leverage a range of participant panels and qualitative research methods to gain feedback from potential study participants to shape their study designs and even their developmental pipelines [6].

In addition to pragmatic industry drivers, and non-profit efforts, national policymakers are taking notice too. Funders such as NIHR in the UK now require evidence of patient involvement in all funded research [12], publishers such as the BMJ require statements of participant involvement (though actual practice is slow to respond [13]), and perhaps most compellingly, regulators are stirring to motion. The Signorell et al. review was itself commissioned by the Swiss Federal Office of Public Health with a view to informing how the Swiss Federal Act on Research Involving Human Beings might be updated to incorporate participant perspectives [1]. FDA’s recent draft guidance require trialists to improve enrolment of participants from under-represented racial and ethnic populations in clinical trials [14]. Given the disproportionate burden inflicted on these populations by typical barriers to trial participation [15], this seems like a key inflection point for systematizing patient listening initiatives. Beyond mere recommendations and good practice guidance, a public consultation underway by the UK’s Medicines & Healthcare products Regulatory Agency (MHRA) proposes the introduction of new legislation which would make it a *legal requirement* to “involve patients and the public in the design, management, conduct and dissemination of research.” As regulators move to requiring participant involvement in trial design, it may be reasonable to request participant input be included in the results published in clinical trial registries as means of increasing distribution and access. To this we would add the requirement that the insights gathered from such research should be shared publicly in the peer-reviewed literature to the extent possible within the constraints of commercial competitiveness. This may help us avoid a two-tier system of participant insights developing between the academic and commercial spheres. No matter our employer, our participants expect us all to design trials thoughtfully to fit their needs and lifestyle. We have much to learn by listening to patients first.

## Data Availability

This article contains no new data.

## Abbreviations

BMJ: British Medical Journal
CRO: Contract research organization
FDA: Food and Drug Administration
MHRA: Medicines and Healthcare products Regulatory Authority
NIHR: National Institute for Health and Care Research
SPFQ: Study Participant Feedback Questionnaire
